# Determinants of pleiotropy and monotonic gene dosage responses across human traits

**DOI:** 10.21203/rs.3.rs-7547111/v1

**Published:** 2025-11-19

**Authors:** Sayeh Kazem, Kuldeep Kumar, Guillaume Huguet, Josephine Mollon, Thomas Renne, Laura M. Schultz, Emma E.M. Knowles, Worrawat Engchuan, Omar Shanta, Bhooma Thiruvahindrapuram, Jeffrey R. MacDonald, Celia M. T. Greenwood, Stephen W. Scherer, Laura Almasy, Jonathan Sebat, David C. Glahn, Guillaume Dumas, Sébastien Jacquemont

**Affiliations:** 1 Centre de recherche Azrieli, CHU Sainte-Justine and University of Montréal, Canada; 2 Harvard Medical School, Department of Psychiatry, 25 Shattuck St, Boston, MA, USA; 3 Boston Children’s Hospital, Tommy Fuss Center for Neuropsychiatric Disease Research, 300 Longwood Avenue, Boston, MA, USA; 4 Department of Biomedical and Health Informatics, Children’s Hospital of Philadelphia, PA, USA; 5 Lifespan Brain Institute, Children’s Hospital of Philadelphia, and Penn Medicine, PA, USA; 6 Department of Genetics, University of Pennsylvania, PA, USA; 7 The Centre for Applied Genomics, The Hospital for Sick Children, Toronto, Ontario, Canada; 8 Department of Psychiatry, University of California San Diego, La Jolla, CA, USA; 9 Lady Davis Institute for Medical Research, Jewish General Hospital, Montreal, QC, Canada; 10 Gerald Bronfman Department of Oncology, Departrment of Epidemiology, Biostatistics and Occupational Health, McGill University, Montreal, QC, Canada; 11 Department of Molecular Genetics, University of Toronto, Ontario, Canada; 12 Mila – Quebec AI Institute, University of Montréal, Canada

## Abstract

While pleiotropic effects of gene dosage are of particular relevance for comorbidities observed in the developmental pediatric and psychiatric clinic, the biological processes underlying such pleiotropy remain unknown. We developed a new functional burden analysis (FunBurd) to investigate all CNVs, genome-wide, beyond well-studied recurrent CNVs. In ~500,000 UK-Biobank participants, we tested the association between 43 traits and CNVs disrupting 172 tissue or cell-type gene-sets. CNVs affected all traits. Pleiotropy was correlated with genetic constraint and was higher in the brain compared to non-brain functions, even after normalizing for genetic constraint. The levels of pleiotropy, measured by burden correlation, were similar in deletions and loss-of-function SNVs and higher compared to common variants and duplications. Gene sets under high genetic constraint showed less monotonic gene dosage responses across traits. Even in the absence of a monotonic response, we observed a negative correlation between deletion and duplication effect sizes across most traits. Overall, functional gene sets are preferentially associated with a given trait when either deleted or duplicated, but rarely both.

## BACKGROUND

Deciphering functions at the cell and tissue level mediating genetic associations with complex traits and conditions is a central question in human genetics^1–4^. It can offer insights into the molecular understanding of causal processes of diseases^1^. Genome-wide association studies (GWAS) have identified thousands of common variants associated with complex traits^1,2^. While functional genomic studies have shed light on the tissues and cell types disrupted by these variants and how they impact gene expression levels, it remains challenging to link common non-coding variants to genes and corresponding functions^1,4–8^.

In contrast, copy number variants (CNVs) – deletions or duplications of DNA segments – invariably lead to a large decrease or increase in gene expression when a gene is fully encompassed in a deletion or a duplication, respectively^9–12^. Therefore, they provide direct insight into the effects of large changes in gene expression on complex traits. However, studies have been limited to a few of the most recurrent CNVs^11,13,14^ due to statistical power. As a result, our knowledge of the CNV architecture of complex traits and biological functions that are sensitive to gene dosage remains limited.

CNVs are commonly screened in individuals referred to clinics for complex neurodevelopmental disorders and congenital malformations^15–17^. Since most clinical studies have focused on pediatric populations, there is limited understanding of the medical issues that may arise later in life. Coding CNVs are associated with broad pleiotropic effects^10–12,18,19^, yet it remains unclear whether these pleiotropic effects are driven by the multigenic nature of CNVs or by particular genes (and corresponding functions) encompassed in the CNVs. Due to statistical power, previous studies have repeatedly analyzed a small set of the most frequently recurrent CNVs^17,19,20^, which collectively affect only approximately 2% of the coding genome^21^. As a result, our understanding of gene functions sensitive to gene dosage is highly biased.

Previous studies have shown that measures of evolutionary/genetic constraint (such as LOEUF score^22^) explain a significant proportion of the effects of coding CNVs on cognitive and behavioral traits^23–26^. It is, however, unclear if the biological function of genes can inform the effects of CNVs beyond constraint metrics. In particular, it is unknown if the effects of CNVs on traits that are not under genetic constraint can be explained by gene function.

**Figure 1:**
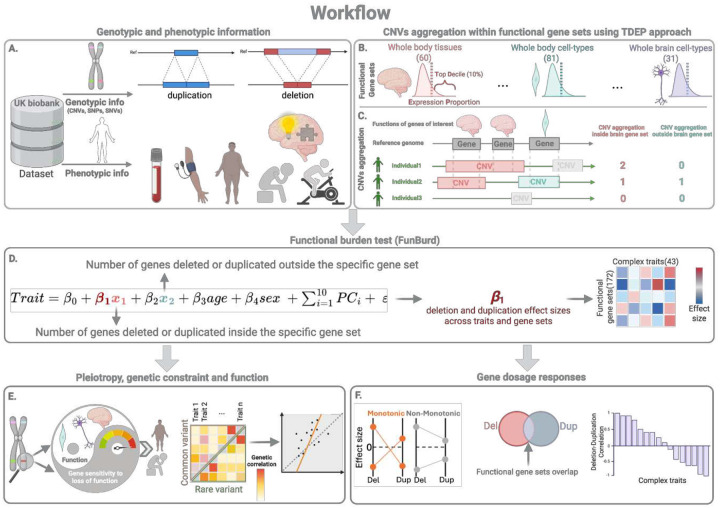
Estimating CNV effects on complex traits and downstream comparative analysis. **A)** We used CNVs and 43 phenotypes from the UK Biobank. **B)** We defined partially overlapping functional gene sets for 60 tissues, 81, and 31 whole body and brain cell types using TDEP (Top Decile Expression Proportion). **C)** CNVs were aggregated within each of the 172 gene sets. **D)** We performed functional burden tests to estimate the associations between CNVs disrupting 172 gene sets and 43 complex traits (IRNT scaled). Tests were performed separately for deletions and duplications. Downstream analyses systematically investigated **(E)** the relationship between gene constraint and function, and rare and common variant architectures of complex traits, and **(F)** monotonicity, correlation, and functional overlap in deletion and duplication effect size profiles. *Figure created with BioRender.*

Our overarching aim is to systematically characterize the CNV architecture of complex traits (both brain and non-brain) across different levels of biological function, encompassing tissue and cell types, and compare it with the common variant architecture. To study all rare coding CNVs (beyond those sufficiently frequent to establish locus-level association), we implemented the functional burden association test (FunBurd)^26^, which aggregated CNVs that disrupt genes with similar functions, defined through shared expression patterns across tissues and cell types (Figure 1).

We quantified the effect size of biological functions disrupted by CNVs for each trait by identifying genes fully present in CNV regions. We then compared these effect sizes to those of common variants and rare SNVs. Our outcome measures included 43 complex traits from 500,000 participants in the UK Biobank. These traits were categorized into two broad groups: brain-related and non-brain-related. Additionally, they were divided into five specific categories: cognitive metrics, mental health, blood assays, physical measures, and reproductive and activity factors. We, therefore, compared these effect sizes to those comprehensively characterized at the common variant and SNV level^18,20,27–30^. This is, to our knowledge, the first large-scale comparison of gene dosage, rare SNVs, and common variants on multiple categories of traits.

### Pleiotropy is higher for genes assigned to brain tissue

**Figure 2:**
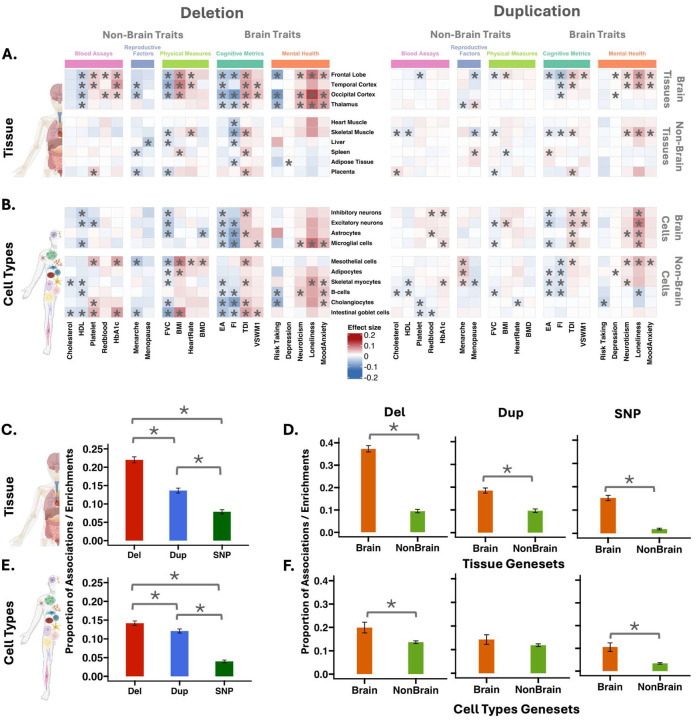
Heatmap of effect sizes for whole body tissue, cell type gene sets across traits. Heatmap displays a representative set of the most significant associations between 5 categories of traits and deletions and duplications aggregated across **(A)** tissues and **(B)** whole body cell types. Traits are categorized and shown along the x-axis, while the y-axis lists gene sets, tissues, and cell types. The blue and red intensity color scale reflects the negative and positive effect sizes. Black asterisks (*) indicate statistically significant associations between traits and genes (FDR correction across 172 ✕ 43 ✕ 2 =14,792 tests). **C-F)** Bar plots (with standard error) summarizing the differences in the level of association/enrichments between the type of variants for **C, E)** all gene-sets, and **D, F)** brain and non-brain functional gene sets. Black asterisks (*) indicate statistically significant proportion differences (FDR-adjusted). **Abbreviations:** HDL: high-density lipoprotein; HbA1c: glycated haemoglobin; BMD: bone mineral density; BMI: body mass index; EA: educational attainment; FI: fluid intelligence; TDI: townsend deprivation index; VSWM: visuospatial working memory; FVC: Forced vital capacity; Del: deletion; Dup: duplication; SNP: single nucleotide polymorphism.

To identify gene-dosage-sensitive functions relevant to tissues across the human body, we performed functional burden analyses to test the association between 43 complex traits (blood assays, physical activity, physical measures, cognitive, mental health measures, and reproductive traits) and 60 gene sets assigned to 60 whole-body tissues which on average showed an overlap of 7.9% (Methods, Figure S1, Table ST5). We chose these 43 phenotypes (Table ST1) relevant to a broad spectrum of medical conditions, because their associations with common variants and rare SNVs have been previously studied^20,27,29^. Out of the 2580 associations (43 traits by 60 gene sets for deletions and duplications separately, Table ST2), CNVs showed FDR-significant association in all 5 trait categories (Figure 2A, Figures S2, and S3). Deletions showed a higher proportion of FDR significant associations than duplications (n-del=568 [22%], n-dup=352 [13.6%]; proportion test p-value=1.2e-14, Figure 2C, Table ST8). Because CNVs are strong contributors to neurodevelopmental and psychiatric disorders^15,16,31^, we stratified functions into brain and non-brain gene sets. The brain compared to non-brain tissue gene sets showed a higher proportion of significant associations (FDR-significant proportion test, Del p-value<1e-16, Dup p-value=2.2e-10, Figure 2D, Table ST9). These findings remained unchanged after removing correlated traits (Figure S4B), demonstrating a higher level of pleiotropy for CNVs affecting the brain compared to non-brain tissue gene sets. Moreover, brain gene sets showed associations with both brain and non-brain traits, underscoring their widespread functional relevance (Figures S2, S3, and S5A). Sensitivity analyses showed that results were not influenced by i) different RNA-seq datasets (GTEx^32^ and Human Protein Atlas^33^, Figures S6-S9); ii) phenotype measures methods, iii) age (<60 versus > 60 years), sex; and iv) ancestries (Figure S10, ST12).

Finally, we asked if these observations were similar for common variants, by performing S-LDSC^5,28^ using the same traits in the same dataset with the same gene sets (Method, Table ST3). While the proportion of significant gene sets was lower (Figure 2C, Figure S11), common variants showed higher levels of association/pleiotropy for the brain compared to non-brain gene sets (Figure 2D), and this remained true when accounting for correlated traits (Figure S4B).

### Gene dosage effects across whole-body and whole-brain cell types

We then asked if differences in pleiotropy observed for tissues were also observed at the cell type level. To do so, we defined 81 gene sets assigned to 81 whole-body single-cell clusters (from Human Protein Atlas, HPA)^34^, with an average overlap of 4.6% (Figure S12, Table ST5). Functional burden analyses showed that 13% of the 6,966 trait / cell-type associations were FDR significant for deletions and duplications (Table ST2). Significant associations were observed across HPA cell type groupings and all 5 trait categories (Figures S13-S14). Deletions showed a higher proportion of significant association than duplications (n-del=494 [14.2%], n–dup=421 [12%]; proportion test p-value=4.8e-3, Figures 2B–E, Figures S13-S14).

Similar to tissue gene sets, the brain compared to non-brain cell type gene sets exhibited a higher proportion of significant associations (Del p-value=5.5e-03, Figure 2F, Table ST8). Beyond pleiotropy, we also observe some level of specificity with brain and non-brain cell type gene sets showing a higher proportion of associations with brain (Del p-value=9.0e-3) and non-brain (Del p-value=1.1e-7) traits, respectively (Figure S5B, Table ST9).

Extending the systematic comparison to common variants using the same cell-type gene-sets showed higher levels of association/pleiotropy for the brain compared to non-brain gene sets (Figures 2F, and S15, Table ST9).

Building on these observations, we focused on human brain cell types, using single-cell RNA-seq data delineating 31 adult brain cell type clusters^35^ with an average overlap of 7.5% (Figure S16, Table ST5). Both neuronal and non-neuronal cell types exhibited pleiotropic effects when disrupted by CNVs (Figures S17-S18, Table ST8). These associations extended to both brain and non-brain traits, with neuronal cell types showing a higher proportion of associations with brain traits than non-neuronal ones, specifically for deletions, suggesting stronger brain-specific relevance of neuronal CNV burden (Figure S5C). For common variants, neuronal gene sets show higher levels of pleiotropy than non-neuronal ones (Figure S19).

### Dissecting pleiotropy, gene function, and genetic constraint

**Figure 3:**
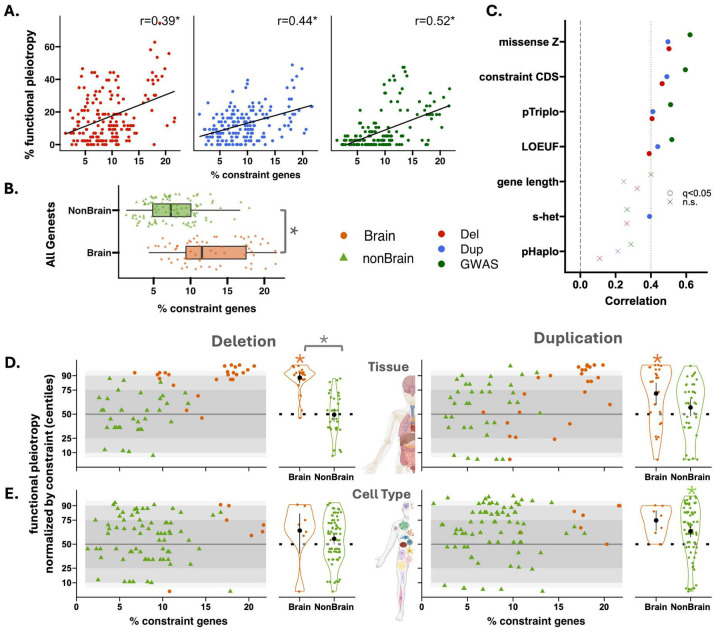
Dissecting pleiotropy, gene function, and genetic constraint. **A)** Correlation between the fraction of constraint genes (LOEUF top-decile) within a gene set and the number of traits showing significant associations with that gene set for deletions and duplications. Each data point is a gene set. Y-axis: the percentage of significantly associated traits for a variant (functional pleiotropy); X-axis: the percentage of top decile LOEUF genes within the gene set. * indicate FDR-adjusted P-value Jaccard (1000 nulls). **B)** Box plots show the distribution of constraint gene percentages for all Brain and Non-Brain gene sets (172). Each point represents a gene set, and asterisks (*) indicate statistically significant differences between groups. **C)** Shows the same information on correlations in panel **(A)** for different constraint metrics. q<0.05: FDR-adjusted P-value Jaccard (1000 nulls) are shown with circles; n.s.: not significant. **D)** Functional pleiotropy, normalized by the fraction of genetic constraint at the tissue level, is shown for 27 brain and 33 non-brain gene sets – deletions are presented on the left and duplications on the right. X-axis: represents the proportion of intolerant genes for the different gene sets. Y-axis: functional pleiotropy normalized by genetic constraint (centile). I.e., the 50th centile shows median functional pleiotropy computed across 100 randomly sampled gene sets. Circles and triangles represent brain and non-brain gene sets, respectively. Gray shaded ribbons indicating 25th–75th (ribbon 1), 10th–90th (ribbon 2), and 5th–95th (ribbon 3) centiles. This is followed by violin plots showing the distribution of normalized functional pleiotropy across brain and non-brain traits. Orange and green asterisks demonstrate significantly (FDR-corrected, q < 0.05) increased functional pleiotropy compared to what is expected for a gene set with a comparable fraction of genetic constraint. The grey star shows a significant difference between the brain and non-brain gene sets, normalized functional pleiotropy. **E)** The same analyses at the whole-body cell type level, including 7 brain and 74 non-brain gene sets. **Abbreviations:** CDS: coding sequence; Del: deletion; Dup: duplication; GWAS: genome-wide significant association; LOEUF: the loss-of-function observed/expected upper bound fraction; n.s.: non-significant; pHaplo: the probability of haploinsufficiency; pTriplo: the probability of triplosensitivity; SC: single cell; s-het: the fitness reduction for heterozygous carriers of a loss-of-function in any given gene; SNP: single nucleotide polymorphism.

Previous studies have shown that common and rare variant heritability enrichments are strongest in constrained genes across complex traits^22,27,29,36,37^. We, therefore, asked if levels of pleiotropy observed above were related to genetic constraint. We observed that functional gene sets with higher proportions of genes under constraint (LOEUF top-decile, Methods) showed a higher level of pleiotropy for deletion (r=0.39, FDR corrected p-Jaccard=7.5e-3, Figure 3A), and duplication (r=0.44, FDR corrected p-Jaccard=3e-3, Figure 3A). This was also the case for functional enrichment analysis computed for the same gene sets and traits using common variant GWAS summary statistics (r=0.52, FDR corrected p-Jaccard=2.4e-2, Figure 3A). This remained true regardless of the constraint metric (Figure 3C, Table ST10). Overall, brain gene sets showed significantly higher proportions of genes under constraint compared to non-brain gene sets (Wilcox Ranksum test p-value=3.5e-12, Figure 3B). Using an approach normalizing functional burden association for genetic constraint (Method section, normative constraint modeling), we demonstrate that the most functional gene sets (~90%) exhibited trait associations within an expected range of 5th to 95th percentiles (Figure 3D, Table ST4). However, several gene sets exhibited significantly higher levels of pleiotropy than expected based on the proportion of genes under constraint (Wilcox Ranksum test, FDR corrected p-value < 0.05, Figure 3D,E). Overall, even after accounting for genetic constraint, the brain compared to non-brain gene sets showed higher levels of pleiotropy (Wilcox Ranksum test, FDR corrected p-value=2.1e-11, for Tissues, Figure 3D).

### Quantifying functional pleiotropy by CNV-burden correlations

**Figure 4:**
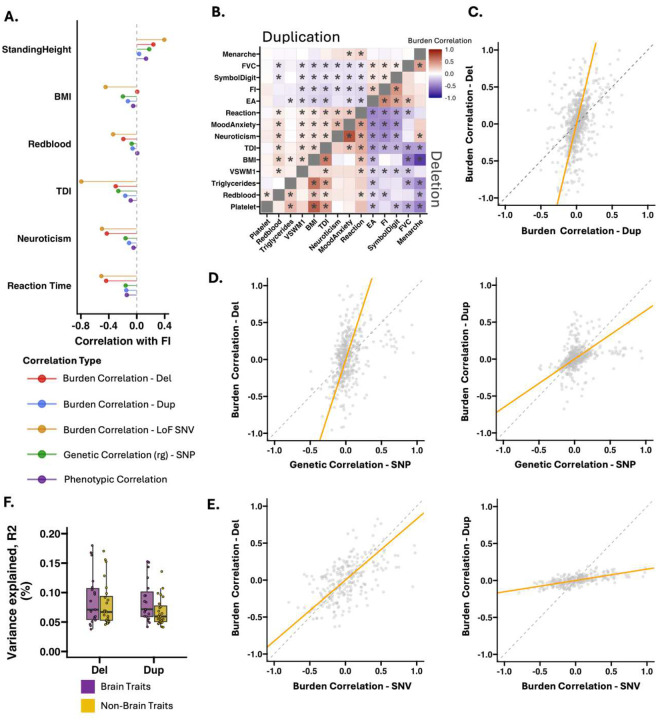
Rare and common variant architectures across complex traits. **A)** Example of genetic and phenotypic correlations between fluid intelligence (FI) and 6 traits, with each point color-coded by correlation type: SNP-based genetic correlations, deletion, duplication burden correlations, loss-of-function SNV burden correlation, and phenotypic correlations. **B)** The heatmap of burden correlations, for deletions and duplications in the lower and upper triangle matrices, respectively. Significant correlations are marked by asterisks (FDR corrected p-Jaccard). **C)** The scatter plot compares effect size correlations between deletions and duplications, where orange lines represent the Total Least Squares regression. The dashed line indicates perfect concordance (y = x). We provide the same comparison between CNVs and **(D)** SNP-based genetic correlations and **(E)** SNV burden correlations; phenotypic correlations are shown in **Figure S20**. **F)** Boxplots illustrate the variance explained by gene sets for both brain and non-brain traits, based on regression analysis. **Abbreviations:** BMI: body mass index; EA: educational attainment; FI: fluid intelligence; TDI: townsend deprivation index; VSWM: visuospatial working memory; FVC: Forced vital capacity; Del: deletion; Dup: duplication; SNP: single nucleotide polymorphism; SNV: single nucleotide variants; LoF: loss of function.

Studies often quantify pleiotropy and shared biological mechanisms^27,38^ through genetic correlation between pairs of traits. This approach has been widely used for common variants and recently extended to Loss of Function single-nucleotide variants (LoF SNVs)^27^, but has not yet been investigated for CNVs. We estimated the CNV-burden correlation between pairs of traits, separately for deletions and duplications, and tested their concordance with each other, as well as against previously published single-nucleotide polymorphism (SNP)-based and rare LoF SNV-based genetic correlations^27^ (Figure 4A, ST7).

Overall, the between-trait burden correlations were concordant across all classes of variants (Figure 4A). The sign concordance between types of variants ranged from 65% to 74% across all traits, which shows the consistent directionality of the between trait genetic correlations across types of variants (Table ST11). Deletion burden correlations were higher than those observed for duplications (4-fold, Figures 4B–C, and S21), and SNPs (2.9-fold, Figure 4D), and comparable to those observed for LoF SNVs (0.82-fold, Figure 4D). In contrast, duplication burden correlations were the lowest among all classes of variants (SNPs: 0.66-fold, Figure 4D, 0.15-fold, Figure 4E, Table ST11). These results suggest that deletions show high levels of pleiotropy, in with previous observations for LoF SNVs. Of note, variance explained estimates for CNVs were small (Figure 4F, Table ST6) and could impact the accuracy of CNV burden correlation estimates.

### Gene dosage responses and deletion-duplication correlations across traits

**Figure 5:**
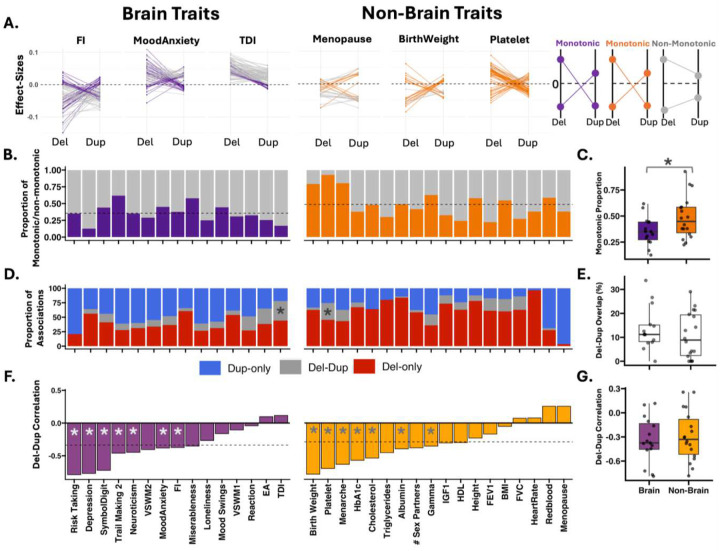
Gene dosage responses across traits. **A)** Illustration of gene dosage responses across 3 brain and non-brain traits. Each line connects the burden correlations of deletions (left) and duplications (right) for the same gene set and trait. Dark purple lines represent monotonic responses (defined as significant effect sizes with opposing directionality for deletions and duplications) for a given functional gene set and a given brain trait. Dark orange shows the same information for non-brain traits. Grey lines represent a non-monotonic response. Only FDR significant effect sizes (for either deletions or duplications or both) are represented. Y-axis: effect sizes. **B)** Summary of gene dosage responses across traits. The proportions of monotonic and non-monotonic responses are shown across brain and non-brain traits.. The total number of responses includes only FDR-significant effects (for either deletions, duplications, or both), and the proportions of monotonic versus non-monotonic responses are calculated within this significant subset. **C**) Box plots depict the distribution of monotonic responses for brain and non-brain traits; * indicates statistically significant (Wilcox Ranksum) differences between groups at the trait level. **D**) Proportion of gene dosage responses significant for deletions-only, duplication-only, and both deletion-duplication, across brain and non-brain traits. **E**) Brain and non-brain traits showed similar levels of deletion-duplication association overlap. **F**) Deletion-duplication effect size correlations across traits. The * indicates significant correlation (FDR corrected p-Jaccard). **G**) Brain and non-brain traits showed similar levels of deletion-duplication effect size correlations.

Deletions and duplications represent a unique paradigm for comparing two classes of variants with opposing effects on gene expression^9^. While monotonic gene dosage responses (i.e., deletions and duplications have opposing effects on a trait) have been observed for some traits at specific genomic loci^39^, it has been challenging to characterize those at the phenome-wide and genome-wide level^40,41^. We used functional burden to characterize 7396 gene dosage responses (172 functional gene sets by 43 traits). We considered 1916 responses that showed an FDR significant association for either deletion or duplication, or both. The proportion of monotonic (i.e., opposing effect sizes for deletions and duplications, Table ST6) and non-monotonic responses was 41.7% and 58.3% respectively. We show that gene sets under high genetic constraint show less monotonic gene dosage responses (r=-0.39, p-Jaccard=1e-3, Figure S22A). Brain gene sets showed less monotonic gene dosage responses compared to non-brain gene sets (Wilcox Ranksum test p-value=1.1e-2, Figure S22B), and reciprocally, brain traits showed less monotonic gene dosage responses compared to non-brain traits (Wilcox Ranksum test p-value=3.5e-2, Figures 5A–C).

However, only 4% and 11% of responses were truly monotonic or non-monotonic (i.e., both deletions and duplications showing FDR significant associations). Most gene dosage responses (85.5%) were undetermined, with significant associations between functional gene sets and traits observed for either deletions (60%) or duplications (40%) and rarely both (Figure 5D Table ST6). Most traits (84%) showed negative deletion-duplication effect size correlations, suggesting that associations between functional gene sets and traits are, in most cases either sensitive to deletions or duplications (Table ST6).

We reasoned that the differential sensitivity of functional gene sets to either deletions or duplications may extend to other classes of variants. We therefore quantified the overlap between gene sets with CNV associations and common variant enrichments across the same 43 traits. Six brain traits showed significant functional gene sets (permutation-based, Figure S24) overlap between common variants (S-LDSC enrichment analysis^5,28^) and duplications (Figures S23A-C, Supplement Results). We did not observe any significant overlap between deletions and common variants (Figure S23B), despite the fact that deletions show a higher number of significant associations with traits compared to duplications (Figures 2C,E, FDR significant proportion test p-value<4.8e-3).

## DISCUSSION

This study represents the first comprehensive investigation into the biological determinants of pleiotropic effects of CNVs and monotonic gene dosage responses. Genome-wide CNV functional burden tests revealed that pleiotropy was correlated to levels of genetic constraint. We also demonstrate higher levels of pleiotropy for the brain, compared to non-brain tissue and cell type gene sets, even after normalizing for genetic constraint. Levels of pleiotropy and shared genetic contributions between pairs of traits were highest for deletions and loss-of-function SNVs compared to duplications and common variants. A minority of gene dosage responses were monotonic, and this proportion was negatively correlated with genetic constraint. While brain traits showed less monotonic gene dosage responses compared to non-brain traits, a negative deletion-duplication effect size correlation was observed across the majority of traits. Overall, brain-related gene sets were under higher genetic constraint, showed higher levels of pleiotropy, and had less monotonic gene dosage responses.

Funburd aggregating CNVs across biological functions demonstrates that rare CNVs genome-wide are associated with a broad spectrum of mental health, cognitive, physical, reproductive, and medical traits (such as blood assays, physical measures, and reproductive factors). Pleiotropic effects were higher in the brain compared to non-brain-related functional gene sets, even with respect to non-brain traits. This provides molecular-level support for the whole body multimorbid presentations in individuals with psychiatric and neurodevelopmental disorders, underscoring that psychiatric conditions share a substantial imprint of poor body health affecting unrelated bodily functions^42–44^. This has important implications for clinical interpretation and management. CNVs are commonly screened in individuals, often with a primary focus on neurodevelopmental disorders^45^, particularly within pediatric populations. Our results suggest that clinical management should be potentially reassessed when individuals transition from pediatric to adult care.

Disentangling genetic constraint from biological function has been challenging, and many studies have highlighted the predominant role of genetic constraint in shaping the genetic architecture of complex traits^22,27,28,36^. Using a novel approach to normalize for genetic constraint, we demonstrate that brain-related gene sets showed higher levels of pleiotropy compared to non-brain gene sets beyond what would be expected for gene sets with similar levels of genetic constraint.

Between-trait CNV burden correlations were the highest for deletions compared to duplications and common variants. This is in line with a previous study showing higher levels of genetic correlation for LoF SNVs compared to common variants^27^. The fact that much of the coding CNV and SNV signals come from genes under constraint may explain why genetic correlations were higher for gene-disrupting variants compared to common variants affecting non-coding regions, as well as a much broader range of genes under less genetic constraint^27,46^. For deletions and duplications with opposing effects on transcription^9^, it has been difficult to observe monotonic effects on complex traits^40^. Our FunBurd approach allowed us to quantify opposing effects of CNVs, showing that non-brain traits have more monotonic gene dosage responses compared to brain traits. This may reflect the fact that the average population value is often the optimal one for non-brain traits, while it is rarely the optimal score for complex brain traits (e.g., adaptive functioning, cognition, educational attainment, mental health, etc.)^40^.

We also demonstrate that gene sets under higher genetic constraint show lower proportions of monotonic gene dosage response. To our knowledge, this has never been reported and may reflect the fact that such genes are intolerant to deletions and duplications (any deviation from normal gene dosage), leading to worse outcomes and non-monotonic responses.

Finally, we showed limited functional overlap between deletions and duplications, with the majority of functional gene sets being sensitive to either deletions or duplications, but rarely both, for a given trait. This suggests that different classes of genetic variation may exert their effects on traits through different biological processes^1,6,8,27^ This led to a negative deletion-duplication burden correlation for the majority of traits, which is more extreme than findings from previous studies showing discordant burden correlations between different classes of rare variant (e.g., missense and LoF variants) for a given trait, also implying that different classes of variants in the same genes often have divergent phenotypic effects^27^.

While functional burden tests address the challenges of rare variant association, limitations of this method include partial overlap between gene sets (similar to LD structure). To address this, we used a new p-value Jaccard method as well as ridge regression to account for overlapping gene sets. The UK Biobank, as a relatively healthy population cohort^47^, poses limitations for disease analysis due to the under-representation of individuals with severe conditions and deleterious CNVs. This bias reduces our ability to detect associations between traits, conditions, and CNVs.

Our functional burden association test provides critical insight into the interplay of genetic constraint, gene function, pleiotropy, and gene dosage response across human traits. We showed that the associations between gene functions and phenotypes are strongly modulated by classes of variants. Gene-centric views aggregating heterogeneous variants within genes may provide an incomplete picture of the biological functions underlying genetic associations.

## RESOURCE AVAILABILITY

### MATERIALS & CORRESPONDENCE

Requests for further information and resources should be directed to and will be fulfilled by the lead PI, Sebastien Jacquemont (sebastien.jacquemont@umontreal.ca).

### DATA AVAILABILITY

UK Biobank data was downloaded under the application 40980, and may be accessed via their standard data access procedure (see http://www.ukbiobank.ac.uk/register-apply). UK Biobank CNVs were called using the pipeline developed in the Jacquemont Lab, as described at https://github.com/labjacquemont/MIND-GENESPARALLELCNV. The final CNV calls are available for download from the UK Biobank returned datasets (Return ID: 3104, https://biobank.ndph.ox.ac.uk/ukb/dset.cgi?id=3104). References to the processing pipeline and R package versions used for analysis are listed in the methods. GWAS summary stats, heritability estimates, and genetic correlations for all the UK Biobank traits are publicly available and were downloaded from the NealeLab: https://www.nealelab.is/uk-biobank. SNV loss of function summary stats and burden correlations are publicly available (Genebass: https://app.genebass.org) and were downloaded from Weiner et al.^27^. Gene-level constraint data are available at https://gnomad.broadinstitute.org and other publications listed in methods.

### CODE AVAILABILITY

The code for generating all the Figures reported in the main analysis and supplement material can be found at the following Github link : https://github.com/SayehKazem/FunBurd

## METHODS

### Participants

The UK Biobank recruited 502,534 individuals (54% female) ages 37 to 73 years, living in the United Kingdom, between 2006 and 2010^48^. Phenotypic measures were collected at the UK Biobank assessment centers (using touchscreen devices), or in an online follow-up. UK Biobank procedures contributing to this work comply with the ethical standards of the relevant national and institutional committees on human experimentation and with the Helsinki Declaration of 1975, as revised in 2008. Data was released under application number 40980. The ethics board of CHU Sainte Justine Research Center, Montreal, Canada, approved this study.

### Genotyping and CNV calling

UK Biobank DNA from blood samples was genotyped using the UK BiLEVE Axiom (n=49,950, 820k probes) and UK Biobank Axiom arrays (n=438,427, 807k probes)^49^. Quality control and processing followed our previously published pipeline^24,26,50^. 733,256 shared, QC-passed, hg19-mapped biallelic probes were used. Samples with high missingness (mind>0.05, |waviness factor|<0.05, log R ratio SD<0.35, B allele frequency SD<0.08) were excluded (n=28,522), leaving 459,855 individuals. CNVs were called using PennCNV^51^ and QuantiSNP^52^, combined with CNVision^31^, and concatenated with CNVs INHERITANCE ANALYSIS (CIAv.2.0), following our published pipeline (https://martineaujeanlouis.github.io/MIND-GENESPARALLELCNV/#/)^24,26,50,53^. CNVs were called using both algorithms with the following parameters: number of probe coverage per CNV ≥3, CNV size ≥1Kb, and confidence scores ≥ 15. CNVs detected by both algorithms were combined according to their types using CNVision to minimize the number of potential false discoveries. Following the data merging steps, CNVs were concatenated using the CNVs INHERITANCE ANALYSIS (CIAv.2.0) algorithm using the following criteria: a) CNV gapping ≤150 kb; b) CNV size ≥ 1000 bps; and c) number of probes ≥ 3. CNVs were filtered according to previously published studies^50^. We kept CNVs passing inclusion criteria such as confidence score ≥ 30 (with at least one of the detection algorithms), size ≥ 50 kb, and unambiguous type (deletions or duplications). All recurrent CNVs were verified visually.

CNVs were annotated using Gencode version 35 lifted to hg19 coordinates (https://www.gencodegenes.org/human/release_35lift37.html). We used bedtools intersect (https://bedtools.readthedocs.io/en/latest/) to identify breakpoints overlapping coding DNA elements such as UTRs, start and stop codons, exons, and introns^24,26,50^.

### Gene-sets

Gene sets were defined based on expression patterns across tissues and cell types. We used three resources:

- Whole-body tissue expression data from FANTOM (Functional Annotation of the Mammalian Genome)^54^ – FANTOM provides whole-body normalized gene expression data (normalized Transcripts Per Million) across 46 tissues. For sensitivity analysis, we used two independent whole-body tissue expression data: GTEx^32^ and the Human Protein Atlas^34^ (https://www.proteinatlas.org/about/download; version 23).
- Whole-body single-cell data from the Human Protein Atlas (HPA)^33^, which provides expression profiles across 81 cell types from 31 human tissues, based on single-cell RNA-seq (scRNAseq) data. In our analysis, we used the average expression data for 81 cell-type superclusters across protein-coding genes (https://www.proteinatlas.org/about/download; version 23).
- Whole-brain single-cell data from the Human Brain Cell Atlas v1.0 (snRNAseq)^35^, which consists of 3.369 million nuclei successfully sequenced using snRNAseq across 106 anatomical locations in adult brains. We used cell types from 31 superclusters and 461 clusters, aggregated data from (https://github.com/linnarsson-lab/adult-human-brain).

### Gene-set creation

We use the Top Decile Expression Proportion (TDEP) approach^28,30^ to create tissue and cell-type gene sets. To do so, we divided each gene’s expression in a given tissue or cell type by its total expression across all tissues or cell types, producing values between 0 and 1. This metric identifies the proportion of a gene’s total expression attributed to a specific tissue or cell type. We then selected the top decile of these expression proportion measures to create each gene set.

### Traits preprocessing and normalization

We extracted 43 binary and continuous complex traits from the UK Biobank (UKBB, Table ST1). For traits with multiple measures over time, we only considered data from the initial visit and assessment. Individuals with missing trait measures were removed from the dataset. We excluded outliers by considering values beyond ±6 standard deviations (SD). To be consistent with previous GWAS for the same UKBB continuous traits, we used PHESANT^55^, an inverse rank normalization transformation (IRNT). This method involves ranking the continuous data and then transforming these ranks into quantiles of the standard normal distribution^56^.

### Functional Burden Association Test (FunBurd)

FunBurd is designed to test the association between variants aggregated across a gene set and a given trait. The traits of interest were considered as a function of the number of genes within the gene set disrupted by CNVs. To avoid effect size inflation, due to multigenic CNVs, we adjusted for the number of genes (not members of the gene set) disrupted by the same CNV. We also adjusted for age, sex, and ancestry.

$$\text{Trait}=\beta_{0}+\beta_{1}x_{1}+\beta_{2}x_{2}+\beta_{3}age+\beta_{4}sex+\sum_{i=1}^{10}PC_{i}+\varepsilon$$

The coefficient B_1_ represents the effect size of a given gene set on the scaled trait(IRNT) of interest. In this analysis, we examined 14,792 associations, derived from 172 gene sets, 43 traits, and 2 types of variations (deletions and duplications). Our group has recently explored similar burden analysis models for the analysis of a single trait^26,57–59^.

### Multiple comparison correction

All analyses were corrected for multiple testing using the False Discovery Rate (FDR). The FunBurd considered the entire matrix, which includes all merged deletion-duplication events and gene sets, totaling 14,792 associations.

### Functional Pleiotropy

“Functional pleiotropy” was defined as the number of traits significantly associated with a functional gene set.

### Permutation preserving Jaccard distance (P-Jaccard)

Because gene sets partially overlap, correlations between gene sets may be inflated. To adjust the p-values of such correlations, we developed a method based on the Jaccard distance matrix. This approach performs permutations conditioned on the Jaccard distance of the gene sets and thus avoids inflated p-values by generating a plausible null distribution^60–62^ (the corresponding Jupyter notebook is available on GitHub).

### GWAS summary statistics, heritability estimates, and genetic correlations

We included the GWAS summary statistics for the same traits derived from nearly the same set of participants from the UK Biobank. GWAS summary statistics, heritability estimates, and genetic correlations for all the UK Biobank traits are available at https://www.nealelab.is/uk-biobank.

### Comparing functional overlap between CNVs and common variants

To systematically compare functional enrichments between CNVs and common variants, we used the same gene sets and UKBB traits to run functional burden association tests (FunBurd) and Stratified LD Score Regression (S-LDSC)^5^. S-LDSC assesses whether certain biological functions are enriched for common variant GWAS signals^28^.

Functional overlap between CNVs and SNPs was defined as the proportion of gene sets enriched jointly in CNVs (FunBurd) and common variant associations (S-LDSC). The Functional overlap between deletions and duplications was the proportion of gene sets associated with both types of CNVs. Significance was tested by fixing the gene sets enriched in common variants and permuting CNV-associated gene sets 1000 times. We then tested whether the observed functional overlap was greater than expected by chance (null distribution).

### CNV- burden correlations

To compute CNV-burden correlations, we used the geneset-trait association profiles of deletions and duplications. Prior to this, we addressed two key issues: the overlap between gene sets and the heritability (variance explained) attributable to functional gene sets. To address gene set redundancy, we integrated two complementary analytical approaches: pairwise Jaccard distances (Figure S1-S12-S16) to quantify overlap among gene sets, and LASSO (Least Absolute Shrinkage and Selection Operator) regression to perform variable selection and regularization. The LASSO model ran on scaled (IRNT) residuals after regressing out the covariates (Sex, Age, and ancestry (PCs) from the scaled trait:

$$\text{Trait}=\alpha 0+\sum_{i=1}^{10}\alpha_{i}\cdot PC_{i}+\alpha_{11}\cdot Age+\alpha_{12}\cdot \text{Sex}+\varepsilon \;r=\text{Trait}-\hat{\text{Trait}}\;r=\beta 0+\sum_{j=1}^{172}\beta_{j}\cdot G_{j}+\varepsilon^{\prime }$$

By considering these two measures, we excluded the gene sets that had a Jaccard similarity percentile greater than 0.2, that are also removed by LASSO in at least 80% of traits, from further analysis (n = 13; see Figure S25). We use selected gene sets (159 out of 172) to calculate the variance explained by these gene sets for each trait (the model is applied on residuals after regressing out the covariance):

$$r=\gamma_{0}+\sum_{k=1}^{159}\gamma_{k}\cdot G_{k}+\varepsilon^{\prime \prime }$$

We then extracted the coefficient of determination (R^2^) from each regression model as a proxy for trait heritability (h^2^ or variance explained).

Finally, we computed deletion and duplication burden correlations between trait pairs using a genetic correlation formula:

$$r_{g}\left(T_{1},T_{2}\right)=\frac{\text{Cov}\left(T_{1},T_{2}\right)}{\sqrt{h_{T_{1}}^{2}\cdot h_{T_{2}}^{2}}}$$

The numerator was defined as the covariance between shrinkage adjusted effect size profiles (excluding the 13 filtered gene sets), while the denominator represented trait specific heritability, estimated as the variance explained by effective gene sets using a linear regression model.

For binary traits, we first applied logistic regression to regress out covariates. The resulting residuals were then treated as continuous outcomes in downstream analyses to compute variance explained (the denominator). Moreover, to enable meaningful comparison of effect sizes and their covariances across binary–binary, binary–continuous, and continuous–continuous trait pairs (the numerator), we transformed the binary trait effect sizes to the liability scale. This transformation ensures that all effect sizes are on a comparable scale:

$$\beta_{\text{liability}}=\beta_{\text{logistic}}\cdot \left(\frac{\phi (t)}{K(1-K)}\right)$$

**β**_logistic_ is the logistic regression coefficient, K is the population prevalence of the binary trait, t is the threshold on the standard normal distribution corresponding to the prevalence, and ɸ(t) is the standard normal density evaluated at t.

### LoF SNV summary statistics of burden correlations

SNV loss of function (LoF) summary stats and burden correlations were downloaded from https://pmc.ncbi.nlm.nih.gov/articles/instance/10614218/bin/NIHMS1933335-supplement-Supplementary_tables.xlsx^27^. Notably, these results were derived from the same set of UK Biobank participants^27^.

### Genetic constraint metrics

We used several measures of constraint, including: i) the loss-of-function observed/expected upper bound fraction (LOEUF) score^22^; ii) probability of being loss-of-function intolerant (pLI)^22^; iii) missense Z-score^22^; iv) CDS: percentage of coding sequence (CDS) under evolutionary constraint^36^; v) two measures of dosage sensitivity^63^: probability of haploinsufficiency (pHaplo), and probability of triplosensitivity (pTriplo); vi) s-het^64^, a constraint metric predicted from multiple transcriptomic and gene features using machine learning; and vii) gene-length^22,63^ in base pairs. We calculated a mean statistic of these measures of constraint for each of our 172 gene sets. To do this, we used genes that overlapped with the top decile (10%) of genes for each constraint measure^22^.

### Normative constraint modeling

We developed a normative model to evaluate whether the association between functional gene sets and traits is explained by genetic constraint. We generated a null distribution by randomly sampling gene sets 100 times (n = 1,298 genes; without replacement) and using different proportions within constraint (top decile of LOEUF scores) and remaining (other LOEUF score deciles), then calculated the corresponding number of trait associations (see Figure S26 for a summary of the implementation in Narval, Compute Canada). This approach allowed us to define the expected relationship between the number of trait associations and the proportion of genes under constraint. Our normative model revealed a sharp increase in trait associations for gene sets with a constraint fraction exceeding 0.25, highlighting a nonlinear relationship between genetic constraint and pleiotropy. By providing a constraint-normalized effect size for each gene set, this approach offers a more refined interpretation of functional pleiotropy.

## Supplementary Material

SUPPLEMENTAL INFORMATION

Figures S1–S26, Tables S1-S12.

Supplementary Files

This is a list of supplementary files associated with this preprint. Click to download.

- SupplementalTablesST1toST12.xlsx
- SupplementalFiguresS1toS26.pdf
